# Cell Therapy of Vascular and Neuropathic Complications of Diabetes: Can We Avoid Limb Amputation?

**DOI:** 10.3390/ijms242417512

**Published:** 2023-12-15

**Authors:** Bernat Soria, Natalia Escacena, Aitor Gonzaga, Barbara Soria-Juan, Etelvina Andreu, Abdelkrim Hmadcha, Ana Maria Gutierrez-Vilchez, Gladys Cahuana, Juan R. Tejedo, Antonio De la Cuesta, Manuel Miralles, Susana García-Gómez, Luis Hernández-Blasco

**Affiliations:** 1 Institute of Biomedical Research ISABIAL of the University Miguel Hernández, Dr. Balmis General and University Hospital, 03010 Alicante, Spain; 2 Institute of Bioengineering, University Miguel Hernández, 03202 Elche, Spain; 3 CIBERDEM Network Research Center for Diabetes and Associated Metabolic Diseases, Carlos III Health Institute, 28029 Madrid, Spain; 4 Fresci Consultants, Human Health Innovation, 08025 Barcelona, Spain; 5 Reseaux Hôpitalieres Neuchatelois et du Jura, 2000 Neuchâtel, Switzerland; 6 Department of Applied Physics, University Miguel Hernández Elche, 03202 Elche, Spain; 7 Biosanitary Research Institute (IIB-VIU), Valencian International University (VIU), 46002 Valencia, Spain; 8 Department of Molecular Biology, University Pablo de Olavide, 41013 Sevilla, Spain; 9 Department of Pharmacology, Pediatrics and Organic Chemistry, University Miguel Hernández, 03202 Elche, Spain; 10 Hospital Victoria Eugenia, Cruz Roja, 41009 Sevilla, Spain; 11 University and Polytechnic Hospital La Fe, 46026 Valencia, Spain; 12 Cell Therapy Centre, Nottingham Trent University, Nottingham NG1 4FQ, UK

**Keywords:** mesenchymal stromal cells, MSC, diabetic foot, foot ulcer, charcot foot, amputation, Critical Threatening Limb Ischemia

## Abstract

Globally, a leg is amputated approximately every 30 seconds, with an estimated 85 percent of these amputations being attributed to complications arising from diabetic foot ulcers (DFU), as stated by the American Diabetes Association. Peripheral arterial disease (PAD) is a risk factor resulting in DFU and can, either independently or in conjunction with diabetes, lead to recurring, slow-healing ulcers and amputations. According to guidelines amputation is the recommended treatment for patients with no-option critical ischemia of the limb (CTLI). In this article we propose cell therapy as an alternative strategy for those patients. We also suggest the optimal time-frame for an effective therapy, such as implanting autologous mononuclear cells (MNCs), autologous and allogeneic mesenchymal stromal cells (MSC) as these treatments induce neuropathy relief, regeneration of the blood vessels and tissues, with accelerated ulcer healing, with no serious side effects, proving that advanced therapy medicinal product (ATMPs) application is safe and effective and, hence, can significantly prevent limb amputation.

## 1. Introduction

### 1.1. The Global Burden of Diabetes Mellitus

Diabetes mellitus is a chronic metabolic disease characterized by elevated levels of blood glucose (hyperglycemia). Persistent hyperglycemia can lead to serious damage to the heart, blood vessels, eyes, kidneys, and nerves. The number of people with diabetes in the world rose from 108 million in 1980 to 422 million in 2014. Prevalence has been rising more rapidly in low- and middle-income countries than in high-income countries as shown in Table 1. The 2023 global burden of Diabetes Mellitus is significant, with about 537 million people worldwide having diabetes [1,2].

From 2000 to 2019, age-standardized mortality rates from diabetes increased 3% yearly increase mostly in lower-middle-income countries (13%) In contrast, the probability of dying from any one of the four main non-communicable diseases (cardiovascular diseases, cancer, chronic respiratory diseases or diabetes) between the ages of 30 and 70 decreased by 22%. Many people with diabetes develop problems with their feet from nerve damage and poor blood flow. This can cause foot ulcers and may lead to amputation. This article deals with innovative treatments for this unmet medical need: ulcers and amputation of the diabetic foot.

#### Types of Diabetes

- Type 1 diabetes (previously known as insulin-dependent, juvenile or childhood-onset) is characterized by deficient insulin production and requires daily administration of insulin. Prevalence of type 1 Diabetes Mellitus in 2017 was approximately 9 million people; the majority of them live in high-income countries.
- Type 2 diabetes may be due either by insufficient insulin production by the beta cells of the pancreatic islets or by defective action in peripheral tissues (muscle, liver) also called insulin resistance. Combination of both leads to high levels of blood sugar if not treated. Factors that contribute to developing type 2 diabetes include being overweight, not getting enough exercise, and genetics [3].
- Gestational diabetes, in which there is dysregulated glucose levels is at an increased risk of complications during pregnancy and at delivery. These women and possibly their children are also at increased risk of type 2 diabetes in the future.
- Monogenic diabetes, directly linked to the mutation of a gene related with the pancreatic beta cell physiology.

### 1.2. Economic Burden of Diabetes

Direct costs of diabetes represent approximately 10% of the budget of developed countries [2,4]. The 10 countries or territories with the highest total health expenditure due to diabetes (20–79 years) in 2021 (in USD billion) are shown in Table 2. The projected increase in the global burden of diabetes highlights the need for effective interventions to prevent and manage diabetes and their complications. For example, healthcare costs from patients with peripheral artery disease (PAD) and diabetes have an estimated cost of more than 80 billion USD in the US [5].

### 1.3. Complications of Diabetes

**Figure 1:**
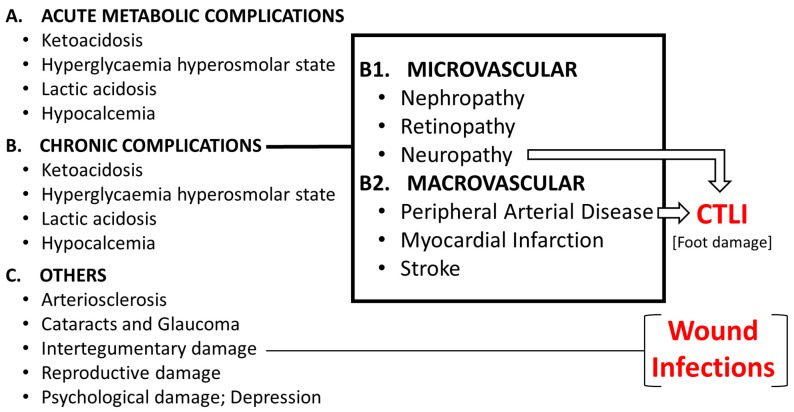
Complications of Diabetes Mellitus. CTLI: Chronic Life Threatening Limb Ischemia (No-Option Chronic Limb Ischemia).

Diabetes can cause a range of complications that affect various parts of the body (Figure 1). In fact, although Acute Metabolic complications are more related with type 1 diabetes, the rest are present in both in type 1 and type 2 diabetes. Both the economic cost and death risk depend on complications. Chronic complications may be classified as: microvascular (retinopathy, nephropathy and neuropathy) and macrovascular (infarction, stroke, peripheral artery disease). Complications are heterogeneous, but un-fortunately, it has not been fully characterized the ethnic, genetic and lifestyle linked to specific complications. hospitalizations, and mortality among diabetic patients and contribute significantly to the high costs of diabetes care (Table 2).

Some of the chronic complications of diabetes include nerve damage (diabetic neuropathy), chronic kidney disease (diabetic nephropathy), eye problems (diabetic retinopathy), foot problems, heart attack, stroke, skin problems, digestive problems, sexual dysfunction, oral and dental problems, etc. These complications can be serious and can lead to amputation, blindness, heart disease, and kidney failure if left untreated. However, with proper management of blood glucose and conventional treatments, many of these complications can be prevented or delayed. This article deals with innovative therapies to treat some of these complications related with PAD, critical limb ischemia (CLI), ulcers and amputations, in fact an unmet medical need. 

About 20% of people with diabetes will develop PAD which remains to be a serious public health problem. Estimated prevalence of PAD in 2019 was 113,443,017 people with an annual incidence of 10,504,092 [6] and 11% of people with PAD may end with Chronic Life Threatening Limb Ischemia (CTLI). 

**Figure 2:**
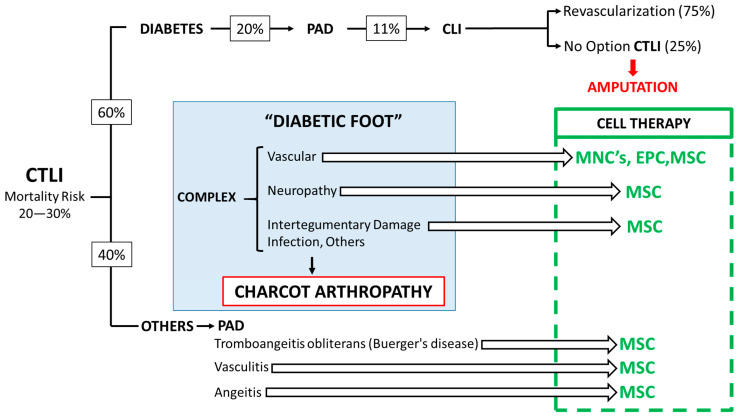
Prevalence and distribution of patients with CTLI. Proposed ATMP treatment depending on the complex nature of the diabetic foot. MNC: Mononuclear Cells, EPC: Endothelial Progenitor Cells, MSC: Mesenchymal Stromal Cells.

Figure 2 summarizes the prevalence and distribution of patients with CTLI (chronic threatening limb ischemia, and CLI with no option of revascularization). Although these figures mostly depend on differences in prevention, evolution and ethnicity. Since there is an estimation of 537 million people with diabetes [2] the risk of CTLI may reach a prevalence of approximately 2.95 million people all over the world (diabetes has a 20% risk of PAD, PAD a 11% risk of CLI and 25% CLI are considered non revascularizable or CTLI).

Since a high percentage of PAD patients develop CLI and 25% of CLI has no option for Revascularization (chronic threatening limb ischemia, CTLI), the risk for ulcers, amputation and mortality increases substantially. Furthermore, the mortality risk associated with CTLI ranges between 20% and 30%. Then, there is an obvious need to develop novel therapeutic strategies focused in ameliorate limb perfusion and neuropathy together with a decrease in the concomitant inflammatory process. Ulcer healing will be a consequence. However, when Charcot arthropathy and infections appears we can only expect a limited response. Although we do not have yet an advanced therapy medicinal product (ATMP) approved by regulatory agencies (FDA, EMA, etc.) to treat CTLI and prevent amputation, the amount of results in pilot, Phase I and Phase II studies strongly suggest that a proper segmentation of CTLI patients and the selection of worth primary and secondary endpoints in the Phase III trials will help to establish the profile for Cell Therapy of CTLI. The right side of the Figure 2 summarizes the ATMP cell therapy options.

#### 1.3.1. The Diabetic Foot

Diabetic foot a complex complication in which PAD, neuropathy, arteriosclerosis and intertegumentary damage are related, with a “personal mix” that differs from one to another patient. Diabetes is a significant risk factor for developing CTLI due to PAD. Patients with diabetes concomitant to CLI represent a sub-group at particular risk. CLI appears when a severe obstruction of the arteries markedly reduces blood flow to the extremities and progress to the point of severe pain and even skin ulcers or sores. Unfortunately, vessels affected in diabetic patients are distal, small diameter and profusely distributed making nonviable surgical revascularization. Furthermore, diabetes is associated with decreased limb survival in patients with CLI.

**Figure 3:**
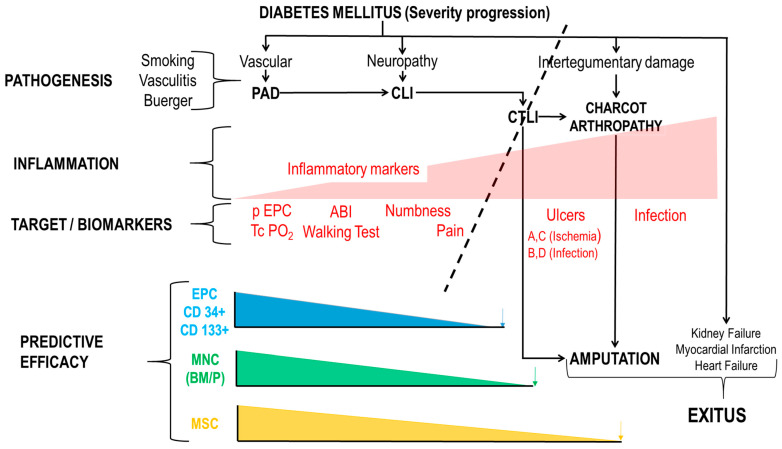
Pathogenesis, target biomarkers and predictive efficacy of AMTPs. PAD: Peripheral Artery Disease. CLI: Critical Limb Ischemia. CTLI: Chronic Life-threatening Limb Ischemia. ABI: Ankle Brachial Index. EPC; Endothelial Progenitor Cells. ABCD; University of Texas Staging System for Diabetic Foot Ulcers. Blue, Green and Orange arrows mean that at that point, outcome is amputation.

Figure 3 also depicts the concomitant process and tentative biomarkers. A decrease in endothelial progenitor cells (EPC) with reduction on oxygen availability (as measured by transcutaneous oxygen pressure-TcPO_2_) and ischemic pain is accompanied by an increase in inflammation and the appearance of ulcers.

CLI characterized by ischemic rest pain, ulcers, or gangrene is the end-stage of PAD, usually associated with a significant risk of affected limb loss and a high risk for cardiovascular events. No-option CLI or CTLI [7,8,9], is an independent risk factor for major amputation in diabetic patients. The annual incidence is approximately 500–1000 new cases per million in industrialized countries [7,10,11]. Then, there are between 0.6 million and 1.36 million people with CTLI per year. According to the World Bank 17% of the world population live in industrialized countries. To note that by 2030, China, Thailand, Mexico and Turkey with join the developed countries, then it is expected that it will be 50% of the world population by 2050. Tobacco, diabetes and age are independent risk factors. Critical threatening limb ischemia grasps for different pathologies: such as Buerger disease, thromboangeitis obliterans and others, but diabetes is in the origin of more than 60% of CTLI. In conclusion, healthcare systems are facing a costly high prevalence threatening situation.

Diabetes is the leading nontraumatic cause of lower limb amputations [12,13]. As a result, neuropathy injuries in their feet and ankles are more frequent and more severe than people without diabetes. Wounds and skin infections heal poorly in persons with diabetes. Endovascular intervention and bypass surgery are the current treatment options [11,14,15,16,17]. However, approximately 20–30% of CLI patients are not eligible for revascularization, or this procedure has failed. 

#### 1.3.2. The Complex Nature of the Diabetic Foot 

Atherosclerosis in distal arteries in combination with diabetic neuropathy can lead to CLI (Figure 1). The distal nature of the arterial narrowing in CLI is less amenable to revascularization. CLI is the advanced stage of PAD, which results from a progressive thickening of an artery’s lining (caused by atherosclerosis) and narrowing of the vessel, which reduces blood flow to the limbs. Patients with diabetes are more likely than other patients to have distal disease that is less amenable to bypass grafting. Limb salvage after bypass is better for insulin and non-insulin diabetics, compared to the endovascular approach.

The pathogenesis of CLI is complex and involves atherosclerosis, peripheral arterial disease, and other vascular risk factors (Figure 3).

#### 1.3.3. Arthropathy of Charcot

Charcot arthropathy (CA), also known as Charcot foot and Charcot neuroarthropathy, affects the bones, joints, and soft tissues in the feet and ankles, caused by an inability to sense injuries, which can result in significant deformities. Diabetes is the main risk factor of CA, but not the only one. Leprosy, syphilis, poliomyelitis, chronic alcoholism, or syringomyelia among other conditions, may also end with CA. It occurs most commonly in patients who have peripheral neuropathy, or loss of sensation, in the foot and ankle, which can be caused by diabetes.

CA results in progressive destruction of bone and soft tissues at weight bearing joints. The neurovascular theory suggests that CA is caused by a combination of vascular and neuropathic factors. The earliest signs of Charcot are mild pain and discomfort, swelling of the foot, redness and warmth that may be confused with and infections and which can occur without an obvious injury.

Hyperglycemia, decreased blood supply and neuropathy that impairs sensation in the feet and makes it difficult to heal wounds, together with pressure and inability to sense injuries ends with minor undetected lesions, deformities and ulcers. Non-surgical conservative treatments (Immobilization, protective footwear, activity changes) are used in the first phases however surgical treatment are necessary with the progression of the disease including debridement of ulcers, exostectomy, deformity corrections, arthrodesis and amputation for severe cases.

One of the key issues is diabetic wounds, particularly leg ulcers and diabetic ulcers. Diabetes slows the healing process by affecting each stage of wound healing, including homeostasis, inflammation, proliferation, and remodeling. This has a long-term detrimental impact on morbidity and mortality as well as quality of life. Diabetic wounds display a long-lasting inflammatory phase together with a delay in the development of mature granulation tissue and a decrease in the tensile strength of the wound. This could be as a result of the ischemia-causing vascular damage [18,19]. In severe cases of CA, it may not be possible to save part or all of the foot resulting in minor or major amputations. 

Prevention of CA and new treatments for this unmet medical need, such as new ATMPs, to avoid amputation are needed. Given the complexity of Diabetic Foot, the results of cell therapy published so far are difficult to compare. 

#### 1.3.4. Treatment of CTLI

##### Conventional Medical Care

Percutaneous transluminal angioplasty (PTA) has become the first line treatment. However, despite the high success rate of PTA, clinical restenosis still represents a huge problem, reaching around 70% at 1-year follow-up [20,21], even more, since in the obstructive pattern is more distal and disperse is quite difficult to succeed with PTA then is considered as no-option CTLI [22,23]. No-option CTLI drives to non-healing ulcers, failure of surgical approaches with a high risk of major amputation, and mortality [24]. 

##### Advanced Therapies: Clinical Trials with ATMP to Treat the Diabetic Foot

In 2001, the EMA (European Medicines Agency) defined Advanced Therapy Medicinal Products (ATMPs)/Advanced Therapies as medicines for human use including Somatic Cell Therapy, Gene Therapy, Tissue Engineering and Combined Advanced Therapies. The legal definitions of Cellular and Gene Therapies can be found in Directive 2001/83/EC amended by Directive Commission 2008/12/EC. Definitions of Tissue Engineering and Combined Advanced Therapies can be found in Regulation (EC) No 1394/2007 [25]. Shown in Figure 2 and Figure 3 are the tentative ATMPs that have been use so far and the expected effects bases in their mechanism of action.

##### Photodynamic Therapies

Typically, the conventional approach for managing diabetic foot ulcers (DFU) involves several key steps, including debridement (cleaning and removal of dead tissue), enhancing blood circulation, maintaining a moist wound environment, and effectively controlling infections [26]. Nevertheless, traditional topical treatments for DFU are expensive and often demonstrate limited effectiveness, especially when faced with multidrug-resistant bacteria, therefore photodynamic therapy (PDT) could stand as a promising approach for addressing infections in the treatment and recovery of DFU. This method entails the application of light-sensitive compounds on the affected area, followed by exposure to either laser (light amplification by stimulated emission of radiation) or LED (light emitting diode) light. When combined with tissue oxygen, this process triggers the generation of reactive oxygen species, leading to a potent local cytotoxic effect that effectively contests the infection in the affected area [27].

### 1.4. End-Points with ATMP Clinical Trials and Mechanism of Action (MoA)

The PAD evolves in stages.

- Stage I: Origin-diabetes mellitus, vasculitis, smoking.
- Stage II: PAD (20% of people with diabetes mellitus). Intermittent claudication.
- Stage III: The combination of PAD and diabetic neuropathy generates chronic life-threatening limb ischemia (CTLI)—intense ischemic pain.
- Stage IV: 25% of CLI cannot be revascularized. “No Option” CLI or CTLI.
- Stage V: Appearance of ulcers.

To note: inflammation and immune system changes are present all over the process.

- Stage VI: CA-PAD. Neuropathy and intertegumentary damage led to CA and the need for Amputation.

Inflammation is important for the initiation and progression of PAD, however, inflammation has been the forgotten factor in PAD, CTLI, and Charcot foot. Figure 3 summarizes the inflammation and immune markers in the process. Even more, when the composition of the multicellular medicament was analyzed in detail (see Section 2.1.2, Bone Marrow Mononuclear Cells (BM-MNCs) and Peripheral Blood Mononuclear Cells (PB-MNCs)) it was observed that not only CD34+/CD133+, EPC were present (1.8–11.5% of the mononuclear cells) but also a high proportion of preregenerative monocytes (6.3%), dendritic cells (1%) and a large proportion of lymphocytes (23.1–47.5%) with CD19+ B-cells (15.7%), CD3+ T-cells (51.6%), CD3+CD4+ T-helper cells (27.4%), CD3+CD8+ cytotoxic T-cells (19%), CD3+CD25+ T-reg (2.5%) and CD3-CD16+CD56+ NK cells (6.3%). We are tempting to speculate that the anti-inflammatory effects of ATMPs in CTLI treatment has been underestimated.

#### 1.4.1. Mechanism of Action

With the previous data and observations, we may propose that ATMP may act by three basic mechanisms:

- Mobilization of endothelial progenitors
- Regeneration of tissues
- Anti-inflammatory effects

##### Mobilization of Endothelial Progenitors

It was already known that peripheral blood EPC were reduced in type 1 and type 2 Diabetes Mellitus [28,29], then we did the most direct and practical approach: take mononuclear cells from the bone marrow and infuse intraarterially into the damaged leg [30,31]. Later on, it was discovered that diabetes impairs hemopoietic stem cells mobilization by alteration of the niche fraction [32], most probably by alterations in the subsets of EPC in bone marrow and spleen [33,34].

##### Regeneration

Neovascularization implies that all of the structures of the arteries, arterioles, capillaries and veins are generated from progenitors and organize themselves properly. An unexpected finding in our first (2007–2009) phase I trial [30] using autologous bone marrow mononuclear cells (see below) was the decrease in insulin needs most probably due to a decrease in insulin resistance due to lower inflammation. 

##### Anti-Inflammatory Effects

It is surprising that inflammatory markers (macrophages, C-reactive protein, IL-1B, IL-6) are not included in the primary and secondary objectives of clinical trials on ATMP for CTLI. We suggest that since inflammation and immune dysfunction is an important component in the progression from PAD to Charcot foot, inflammatory markers should be included as primary and secondary end points. In our experience [30,35], demonstrates that a single dose of ATMPs effects take 3 to 6 months to “reset-regenerate” de diabetic foot and comorbidities, then a longer follow-up may be more significative than results on the first period (3–6 months).

All of these effects contribute to wound healing but cannot recover a finger or the feet since regeneration of members is not possible in adult mammals. Even more, now we know that it takes months (3 to 12) to observe full regenerative and healing process and that it seems difficult to observe short-term effects (0–3 months), furthermore limb salvage has to be clearly defined. Subsequently, amputation during the first 3–6 months after ATMP administration may not be related with ATMP therapeutic effects. In contrast, long-term effects on mortality and amputation have been reported.

#### 1.4.2. Primary and Secondary End-Points in Clinical Trials with ATMP

We here suggest that End-Point should be linked to the MoA and predictive efficacy (Figure 3). Summarizing from Figure 3 the following markers (end-points) should be followed:

- TcPO_2_: Primary
- Peripheral EPC: Primary
- Inflammatory markers: Primary and secondary
  ◦ C-reactive protein
  ◦ Proinflammatory cytokines: TNF-α, IL-6, IL-1B
  ◦ Anti-inflammatory cytokines: IL-4, IL-10
- Inflammasome: Secondary
- Pain-walking test: Primary
- Ulcer healing: Primary and Secondary

Probability of healing without a major amputation during follow-up depends on:

i. Ulcer Infections
ii. Increase in collateral vessels indicating vascular remodeling Revascularization with TcPO_2_ > 40 mm Hg
iii. Keeping kidney function with glomerular filtration rate  >  30 mL/min

Phase I and Phase II studies with ATMP therapies appear to demonstrate improved rates of limb salvage associated with revascularization compared with the results with Standard of care of non-revascularized patients with diabetes, PAD and ulceration previously reported. Long-term mortality rates: amputation rate at 1 year decrease following the first treatment with peripheral-blood mononuclear cells (PB-MNCs) [30]. Then amputation, when included should be a long-term end-point (5–10 years post administration)**.** Amputations (minor or major), especially during the first 3 months after administration of ATMP, are the consequence of a complex process that began long before and also depends on other factors (infection, intractable ischemic pain, bone and tissue damage, etc.), however note that long-term (1–5 years) prevention of amputation has been observed.

All of the above provide the rationale for the use of advanced therapies for no-option patients, such as implanting autologous MNCs (that can be isolated from the bone marrow or the peripheral blood); either autologous or allogeneic Mesenchymal Stromal Cells (MSC) or EPCs, (including CD34+ and CD133+). These procedures have shown to be safe and effective, as indicated by a series of randomized and not randomized Phase II clinical trials [36]. However, the number of completed and published Phase III clinical are still very limited [37]. The heterogeneity in terms of the cell types and cell subsets within the cell preparation, method of cell injection, and the lack of a clear definition of the associated MoA call for new studies. 

In summary, stages I to V can be treated by Cell Therapy whose MoA leads to Neovascularization with increased tissue oxygenation (TcPO_2_), decrease in inflammation and recovery from tissue damage. These objectives can be included as “Primary End-points”-since they can be improved by the therapeutic action of a medication.

## 2. Results: Clinical Trials with ATMPs

### 2.1. Clinical Trials with ATMP of CTLI in Diabetic Patients

Treatments of PAD are focused on improving perfusion and oxygenation in the affected limb. Standard revascularization methods include bypass surgery, endovascular interventional procedures, or hybrid revascularization. Cell-based therapy can be an alternative strategy for patients with no-option CLI who are not eligible for endovascular or surgical procedures. Table 3 summarizes the different approaches to treat CTLI carried out in Spain. 

Numerous stem and stromal cell populations from various sources have been proposed for cell-based therapy: both bone-marrow and peripheral mononuclear cells, MSCs, EPCs and their products may play a pivotal role in therapeutic neovascularization and treatment of limb ischemia. Then, in cases where conventional treatment options have been exhausted and there is no possibility of further revascularization for the affected limb, cell therapy is now suggested as an experimental therapeutic approach in clinical trials for individuals dealing with severe forms of PAD [38]. Contraindications for this approach encompass several factors. These include an anticipated patient survival of less than six months, the presence of a known bone marrow disease (such as lymphoma, leukemia, myelodysplastic syndrome, or metastatic bone marrow impairment), chronic renal insufficiency requiring dialysis therapy, and acute limb ischemia accompanied by a severe inflammatory response that poses a life-threatening risk, necessitating early limb amputation [39].

**Table 3:** Spanish effort to treat CTLI with ATMP. ATMP: Advanced Therapies Medicinal Product, MNC: Mononuclear Cells, EPC: Endothelial Progenitor Cells, MSC: Mesenchymal Stromal Cells. Registered in ClinicalTrials.gov. Route Intraarterial, CRD: Clinical Research Document.

Product	ClinicalTrails.gov (NCT, First Posted-IATA FPS)	Mechanism of Action	References
BM-MNC (autologous)	Phase I NCT00872326 First Posted IATA-FPS: 30 March 2009	1—Mobilization of EPC DM 2—Regenerative Cytokines 3—Inflammation and Immune	Completed [30,31] Soria et al., 2023 (this work)
BM-MNC (autologous)	Phase I NCT00987363 First Posted IATA-FPS: 30 September 2009	1—Mobilization of EPC DM 2—Regenerative Cytokines 3—Inflammation and Immune	Completed [40]
BM-MNC (autologous)	Phase I-II, Multicentric NCT014008381 First Posted IATA-FPS: 30 August 2011	1—Mobilization of EPC DM 2—Regenerative Cytokines 3—Inflammation and Immune	Completed No publications
Adipose-derived MSC-Diabetes (autologous)	Phase I-IIa NCT01257776 First Posted IATA-FPS: 10 December 2015	2—Regenerative Cytokines 3—Inflammation and Immune	Completed [35,41,42] Soria et al., 2023 (this work)
Adipose-derived MSC-No Diabetes (autologous)	Phase I-IIa NCT01745744 First Posted IATA-FPS: 10 December 2012	2—Regenerative Cytokines 3—Inflammation and Immune	[35,41,42] Soria et al., 2023 (this work)
Endothelial Progenitor Cells (autologous)	NCT02287974 First posted IATA-FPS: 10 November 2014 Last: 19 December 2018	1—Mobilization of EPC DM 2—Regenerative Cytokines 3—Inflammation and Immune	Recruitment closed by sponsor No publications
Adipose-derived MSC-(allogenic)	Phase II, Multicentric NCT04466007 First Posted IATA-FPS: 11 January 2021	2—Regenerative Cytokines 3—Inflammation and Immune	Recruitment completed [43] No data yet, CRD to be opened in 2024.

#### 2.1.1. Regulatory Requirements

As of 21 July 2014, it became mandatory for sponsors to post clinical trial results in the European Clinical trials Database (EudraCT), managed by the European Medicines Agency (EMA). This date corresponds to the finalization of the programming of the database as referred to in a European Commission guideline, in application of the current clinical trials Directive 2001/20/EC. Sponsors are obliged to post results in EudraCT for any interventional trials registered in EudraCT and that have ended within a certain period of time: For any interventional clinical trials that ended on or after 21 July 2014, sponsors will have to post results within six or twelve months following the end of the trial, depending on the type of trial concerned. For trials that ended before that date, sponsors will need to submit the results retrospectively, in accordance with the specific timeframe laid out in the above-mentioned European Commission guideline on the posting and publication of result-related information on clinical trials [25].

#### 2.1.2. Autologous ATMPs

##### Bone Marrow Mononuclear Cells (BM-MNCs) and Peripheral Blood Mononuclear Cells (PB-MNCs)

Both bone marrow and peripheral mononuclear cells have been tried. Tateishi-Yuyama et al. [44] demonstrated the efficacy of intramuscular infusion of autologous mononuclear cells in CLI. However, people with diabetes were excluded from this trial. From 2007 to 2009 we did a Phase I-II trial in type 2 and type 1 Diabetes Mellitus in a prospective observational study using autologous peripheral blood mononuclear cell therapy for no-option CTLI with promising results [30,31]. Intraarterial administration of freshly prepared autologous bone-marrow mononuclear cells was safe, feasible and the therapeutic dose could be reached with a single intraarterial dose. Since in diabetes there is a substantial decrease and function of peripheral endothelial progenitor cells [28,29,45], most probably due to the lack of neural control of the bone marrow by the diabetic neuropathy, the driving idea was to take these cells from the bone marrow and infuse them intraarterially in the damaged tissues using the intraarterial route. In 2011, we reported the results of a Phase I/II, prospective, single-centre study, with consecutive inclusion of 20 diabetic patients with CLI due to below-the-knee extensive arterial disease observational study on no-option CTLI subjects with diabetes and ischemic foot ulcers that received intraarterial injections of BM-MNCs [30]. Table 4 summarizes the characterization of MNC subpopulations by flow cytometry in Bone-Marrow aspirate as compared with Cord Blood and Peripheral Blood. 

Table 5 describes the Rutherford-Becker Scale patients’ classification (RB), while Table 6 summarizes the results using the R-B scale and the University of Texas criteria for ulcers. To note that R-B categories improve substantially 3 and 12 months after administration. Baseline categories 6 (30%), 5 (55%) and 4 (15%) move to categories 2 (18%), 1 (56%) and 0 (25%) 12 months after administration. Also, ulcers healing, as measured by the University of Texas scale change from stage C (C3: 30%, C2: 15%; C1: 15% and C0: 15%) to stage A (A0: 87.5%, A1: 6.25%).

**Figure 4:**
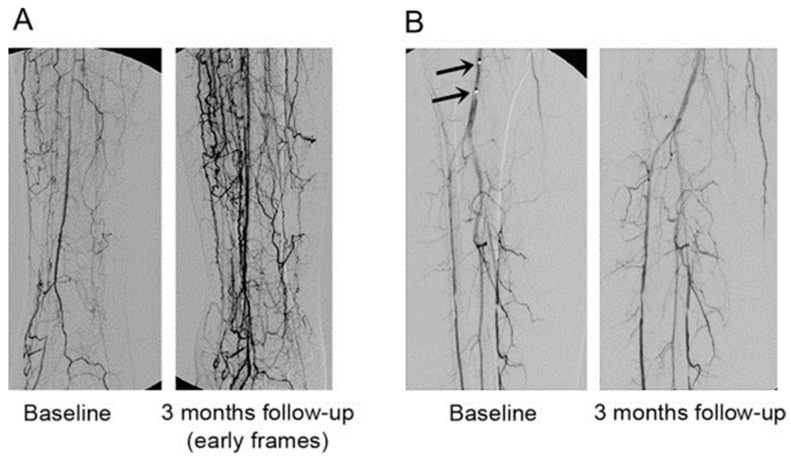
Control Arteriography (Baseline) and 3 months follow-up after intraarterial administration of autologous bone-marrow mononuclear cells (taken from Ruiz-Salmeron et al., 2011, with permission [30]). (**A**) Patient with baseline patency of only peroneal artery presented at three month follow-up an outstanding vascular network growth, with collateral arteries enlargement and denser network due to proliferation of new branches. (**B**) Angiographic image at baseline (left) showing the site of autologous BM-MNC administration (black arrows) and at three months after (right) where new collateralization of capillary vessels was appreciated (taken from Ruiz-Salmeron et al., 2011, with permission [30]).

Diabetic patients with CLI showed improvements in the clinical picture associated with said pathology as ulcer healing; increase in vascularity and consequently in the temperature of the member; increase in TcPO_2_; increase in the Ankle-Brachial Index (ABI); and no patient required major amputation. Unlike other studies, the intra-arterial route was chosen in this trial and the cellular dose was 10 times lower than the dose used by other groups. Also, surprisingly, six of the diabetic patients receiving insulin had a reduced need for it. Is tempting to speculate that BM-MNC have a direct action on insulin production and/or effects, however the most plausible interpretation is that BM-MNCs reduce the inflammation in the peripheral-damaged tissue and subsequently decrease insulin resistance, than a lower dose of insulin is needed (Figure 4).

##### Allogeneic-MSC: Mesenchymal Stromal Cells

**Figure 5:**
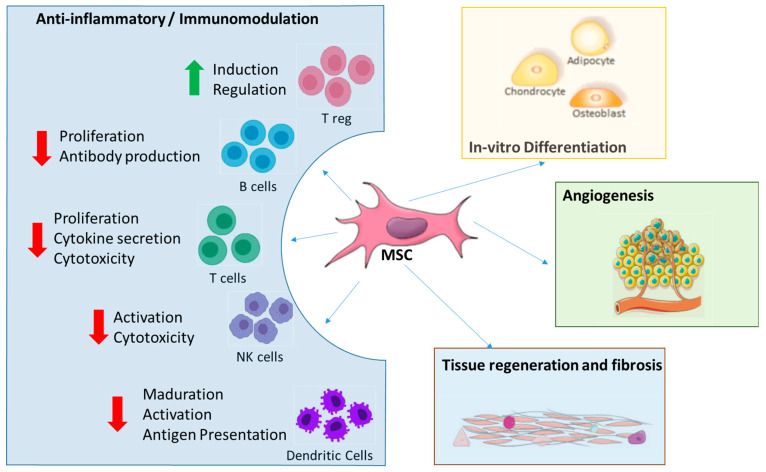
Four main properties of mesenchymal stromal cells (MSC). MSCs have a potential for differentiation into ectoderm cells (neurons, epithelial cells), mesoderm (osteocyte, endothelial cell, chondrocyte, and adipocyte), and endoderm (muscle cells, gut epithelial cells and lung cells). Secretion of factors such as proteins, miRNAs, mitochondria, and exosomes can promote repair of damaged tissue and immunomodulatory potential. The immunomodulatory effect is mainly immunosuppressive, several secreted cytokines inhibit the activity of natural killer cells (NK cells), T cells and B cells; other cytokines activate the proliferation of regulatory T cells (T reg) and the switch from macrophage M1 (pro-inflammatory) to macrophage M2 (anti-inflammatory). The property of migration and homing is possible by the expression of specific ligands and receptors in the site of injury. Red arrows, decreased levels; Green arrow, increased levels.

MSCs are non-hematopoietic cells present in the multiple locations (bone marrow, adipose tissue, umbilical cord, dental pulp and many other tissue sources). MSCs, as stromal cells in the bone marrow, constitute an essential part of the marrow microenvironment supporting hematopoiesis, also possessing extensive proliferative capacity [48], in other tissues play a regenerative role being considered in fact “our own internal cell medicaments). MSC have multilineage potential with the ability to differentiate (Figure 5) in-vitro into adipogenic, osteogenic, chondrogenic, and skeletal muscle cells, as well as into vascular smooth muscle cells, neural precursors, cardiomyocytes, or perivascular cells [49,50,51]. More important for this topic, MSCs are anti-inflammatory, promote angiogenesis and tissue regeneration.

MSCs have garnered significant attention as promising candidates for cell therapy due to their pivotal roles in maintaining tissue and organ balance, facilitating repair, and providing support through self-renewal and multi-differentiation capabilities. Additionally, MSCs are recognized for their diverse range of beneficial properties, including anti-inflammatory and anti-proliferative effects, immunomodulatory capabilities, trophic functions, and the ability to promote angiogenesis [52], by secreting cytokines such vascular endothelial growth factor (VEGF) and basic fibroblast growth factor [53]. Additionally, by migrating to sites of inflammation and altering the phenotypic of dendritic cells (DC), T cells, B cells, and NK cells, they also play a critical role in immunomodulation. They limit DC maturation, downregulate proinflammatory cytokines, escape CD8+ T cell-mediated apoptosis, and decrease T-lymphocyte proliferation through transforming growth factor-beta 1 (TGF-1) and other growth factors such hepatocyte growth factor and nitric oxide. TGF-1 is important for the immunomodulation of MSCs since it increases the generation of regulatory T cells [49].

Both basic preclinical medical research and clinical studies focused heavily on MSC considered to be the “physiological” medicaments that cure the body in adult beings. Initially, it was believed that after being administered, MSCs would move to injury sites, engraft, and develop into functional cells (as happens “in-vitro”), leading to the regeneration of injured or ill connective tissues. In contrast with the initial observations, when the “stem” cell properties such as in-vitro differentiation were considered therapeutically relevant, however MSC are used now on the basis of other properties (immunomodulatory, anti-inflammatory, antiapoptotic, etc). Then, MSCs operate through a variety of mechanisms (Figure 5): (a) differentiation into particular cell lineages and integration into tissues, which have applications for regenerative medicine; (b) secretion of molecules like cytokines and exosomes that support cell growth and survival as well as control inflammation; (c) direct MSC contact with host cells to alter the functions of effector cells; (d) and immunomodulation of immune cell depending on the local microenvironment or disease status [54,55].

Mesenchymal Stromal Cells, due to its unique biological properties including adhesion to plastic, easy expansion and culture, are the cell type mostly used in Cellular Therapy. The minimum criteria for the characterization of human MSC are: (i) adherence to the plastic of the culture plate; (ii) adipogenic, chondrogenic and osteogenic differentiation capacity; and (iii) a specific profile of surface markers CD105+, CD73+ and CD90+, CD45, CD34−, CD14− or CD11b−, CD79a− or CD19− and HLADR− [56]. MSCs are also involved in the regenerative process [57]. Even more, preliminary evidence suggests that secretion of soluble factors could explain most of the beneficial effects of MSCs. They have multiple actions (Figure 5), including support of angiogenesis, modulation of inflammatory and immune reactions, protection against apoptosis, and stimulation of EPCs. MSCs have also been shown to express and secrete factors essential for the process of angiogenesis, such as SDF-1, VEGF, basic fibroblast growth factor (FGF), or matrix metalloproteinases for the process of angiogenesis. MSCs are also able to stimulate endothelial cell migration and tube formation [58]. Moreover, MSCs have a vital role in stabilizing the new vasculature through their role as pericytes. These perivascular cells control proliferation and migration through interactions between endothelial cells [59]. 

Summary of Results of Intraarterial administration of adipose tissue-derived mesenchymal stromal cells on CTLI.

These results were previously published as part of the PhD Thesis (in Spanish) of Dr. N. Escacena [35]. We assayed autologous MSC from the bone-marrow in a Clinical Trial approved by the Spanish Agency for Medicines and Health Products (AEMPS) and registered in the European Clinical trials Database (EUDRACT) and NIH-US Clinical Trials data bases (CeTMAd/ICPD/2008; EudraCT: 2008-001837-88; NCT01079403). The protocol was approved by the AEMPS in 2009 and was presented and approved by the Clinical Research Ethics Committee (CEIC) of the Virgen Macarena University Hospital in Seville. It has been financed by the Carlos III Institute (ISCIII) within the Strategic Health Action call; State Subprogram for the Generation of Knowledge, Health Research Projects. It was carried out within the framework of collaboration with the following public entities: FPS-CABIMER, the Virgen Macarena University Hospital in Seville and the San Lázaro Hospital in Seville. Following EU Regulation No 536/2014 (which came into application on 31 January 2022), results of clinical trials must be published 1 year after the end of the trial [NCT 01079403]/[EudraCT 2008-019774-33], although this Ph.D. Thesis is in itself a publication [35], open science and European Union Regulations forced us to give access to a summary of the results presented here.

This is a phase I-II, prospective, multicenter, open, randomized and controlled study with parallel groups for 2 dose levels, with the objective of assessing the safety and feasibility of the infusion of mesenchymal stem cells administered intra-arterially, in diabetic patients with chronic critical ischemia of the lower limbs and without possibilities of revascularization or therapeutic alternatives. 

Follow-Up: The study period covered 12 months from the infusion of the AdMSCs to the patient until closure.

##### Neovascularization

There is a significative increase in the extension of vascular branching and vascular density after 12 months when the treated group is compared with the control group. Therefore, we can conclude that throughout the study, 35% of the patients experienced an increase in neovascularization in the infused limb attributable to the cell therapy received. This increase was maintained over time, as it was observed both at 6 months and 12 months after cell infusion. On the contrary, in 45% of the patients the vascularization decreased according to the data provided by the quantification.

##### Ulcer Healing

**Figure 6:**
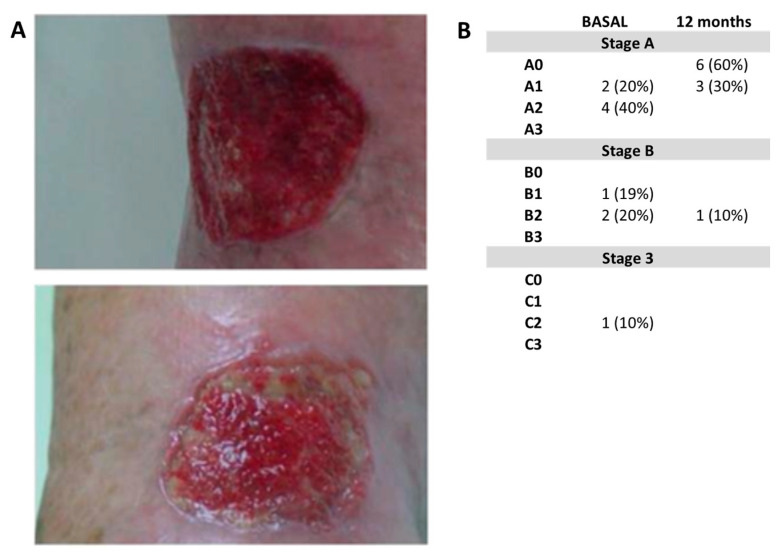
Healing of ulcers. (**A**) Representative image of the healing of an ulcer at baseline and 45 days after cellular treatment, observing good granulation tissue. (**B**) Evolution of ulcer healing and its classification at the Texas Scale (taken from Escacena, 2016 [35]).

Regarding the evolution of ulcer healing, 10 patients (33.3%) presented non-healing ulcers in the target limb at the beginning of the study. Five of these were the result of surgical wounds after amputations suffered prior to inclusion in the trial. According to the University of Texas Classification for diabetic ulcers, one patient had grade C2, two patients had grade B2, one had grade B1, four had grade B2, and two had grade A1. At the end of the study, the Texas scale reflected complete healing of trophic lesions in six cases (A0), except for three patients who presented superficial ulcers (A1) in the first two cases, and one infected ulcer of considerable proportions (9 × 14 cm) in the case of one patient of the control group. Ulcer healing in the two treatment groups with cells was faster in most cases compared to the control group, whose evolution occurred more slowly and discreetly (Figure 6).

Ulcer healing in the two treatment groups with cells was faster in most cases compared to the control group, whose evolution occurred more slowly and discreetly (Figure 6).

##### Pain and Walking Capabilities

**Figure 7:**
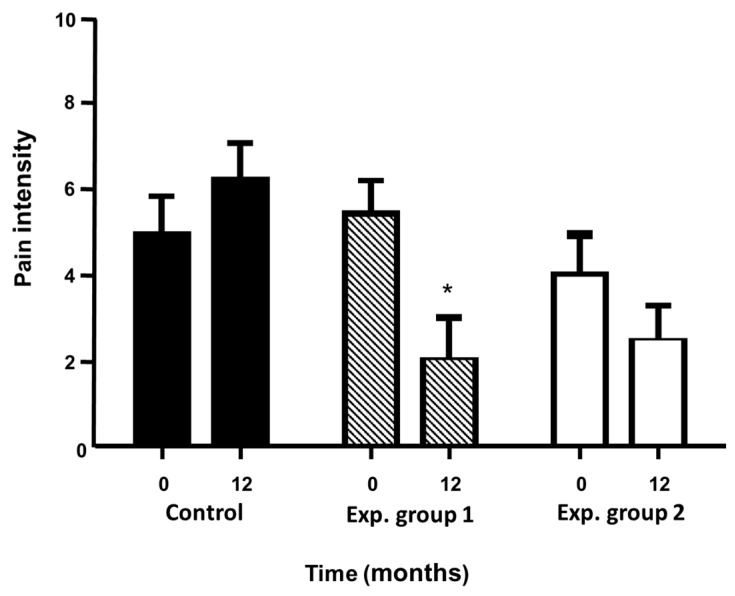
Effects on Pain. Patient evolution on pain intensity over time thorough the essay. Statistical analysis using paired ttest (sig. Two-tailed) * *p* ≤ 0.05 (taken from Escacena, 2016 [35]).

Overall, pain was reduced by 60% of patients in the study, 53.3% (16 patients) belonged to the cell treatment groups and 10 patients of them (33.3%) were asymptomatic in terms of pain at the end of the study, all of them being they from the cell treatment groups. However, to estimate the evolution of the severity of the disease in the study patients, the changes produced in the Rutherford-Becker Scale and TcPO_2_ were analyzed. The comparison between the different groups showed a statistically significant difference both between the experimental group 1 with respect to the control group (*p* ≤ 0.001) as between the experimental group 2 and the control group (*p* ≤ 0.01) as is shown in Figure 7.

##### Transcutaneous Oxygen-TcPO_2_

**Figure 8:**
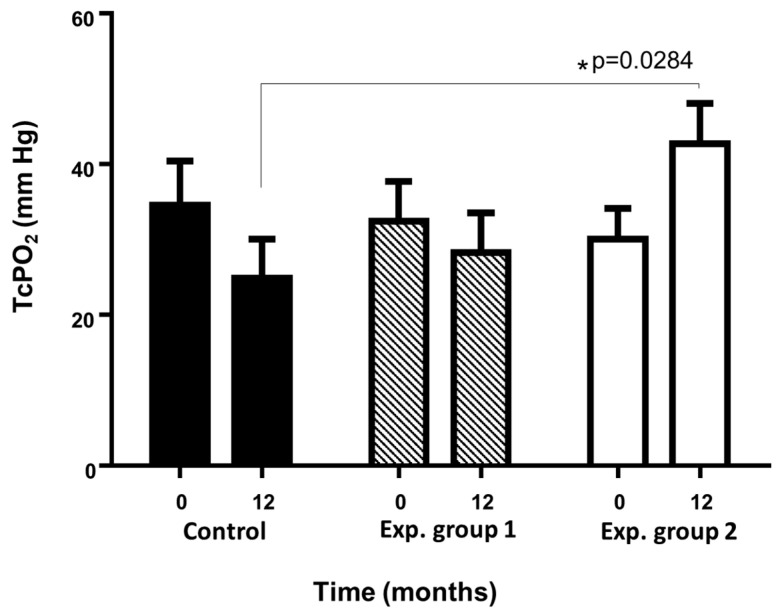
Evolution of patients in transcutaneous oxygen pressure (TcPO_2_) throughout the essay. Statistically significant evolution of the experimental group 2 vs. Control (* *p* < 0.05) (taken from Escacena, 2016 [35]).

The mean of the values presented at the beginning of the trial by the control group and the experimental groups were 34.5 ± 16.56 mm Hg, 32.4 ± 16.81 mm Hg and 30 ± 12.88 mm Hg respectively. At the end of the follow-up (12 months), the collected values were 24.9 ± 16.21 mm Hg, 28.3 ± 16.5 mm Hg and 42.67 ± 16.1 mm Hg, experimental group 2 being the only one that experienced an increase statistically significant (*p* < 0.05) as shown on Figure 8.

##### Ankle-Arm Index (ABI)

Diabetic patients with CLI showed improvements in the clinical picture associated with said pathology as ulcer healing; increase in vascularity and consequently in the temperature of the member; increase in TcPO_2_; increase in the Ankle-Brachial Index (ABI).

##### Rutherford-Becker Scale

The overall rate of improvement for all study patients at 6 months was 40% (Table 7). When the evaluation was carried out separating the grades, for all patients, 42.1% of those categorized as R II-4 improved, while only 36.4% of R III-5-6 did. In treatment group 1 there was a greater improvement in the group categorized as R III-5-6 compared to R II-4 (50% vs. 33.3%). In the treatment group 2 and in the control group, the improvement was greater in patients with grade II-4 (66.7% vs. 50%; 28.6% vs. 0%). It should be noted that in the control group there was no improvement in the patients with the most severe symptoms, that is, those patients classified as R-III. In addition, the improvement in the R-II group of control patients is modest compared to the groups treated with cells (28.6% vs. 33.3% and 66.7%), the control group being the one with the most patients with this graduation had at the beginning of the study (80% vs. 60%).

At 12 months of treatment, the overall improvement in all patients was 60% compared to the start of the study, a percentage that coincides in both grade II and grade III patients. The improvement by treatment groups resulted in 60% in group 1, 80% in group 2 and 40% in group 1. Control group, the latter being clearly lower than the groups treated with cells. With respect to the RB grades, in grade II 37.5% of the subjects improved. Patients in the control group, compared to 50% and 100% of the groups treated with cells 1 and 2 respectively. Patients with grade III on the RB scale, experienced a 50% improvement in the control group that coincides with treatment group 2. In treatment group 1 the improvement was 75% of the patients.

##### Amputations

Five patients had amputations prior to the study. During the year of follow-up, no patient suffered a major amputation or in the target limb or on the contralateral. However, five patients experienced minor amputations at the digital level, three of which presented grade III on the RB Scale. One patient presented amputation in the contralateral limb, while 4 of them presented it in the target limb. Table 8 summarizes the number of patients as well as the percentage of amputations suffered throughout the study (6 and 12 months) in the three randomization groups. One patient suffered four minor amputations in total, the first of which occurred before cell infusion. Four days after the infusion, he required hospital admission due to infection in the amputation wound, with new skin lesions appearing. Three months after the infusion, he presented amputation of the 4th finger. At six months, he presented amputation of two other fingers (2nd and 3rd). At nine months after the infusion, all of the fingers of the target limb have already been amputated, although it presented excellent granulation tissue. In the control group one randomized patient suffered amputations in both limbs, as well as various ulcers (a total of 4) throughout the study. Four fingers of the target limb were amputated.

##### Endothelial Progenitor Cells (EPC)

The number of circulating EPCs, first described by Asahara et al. [60], is small and almost null in diabetes [28,29]. Their number in the peripheral blood increase in response to ischemia by mobilization from the BM after secretion of proangiogenic cytokines, such as the VEGF, Stromal Cell-Derived Factor 1 (SDF-1), or Hypoxia-Inducible Factor 1 (HIF-1) a mechanism that seems to be inefficient in diabetes. Under physiological conditions, EPCs will be addressed and attracted to ischemia sites and contribute to angiogenesis by secreting interleukins, growth factors, and other cytokines by activating resident stem cells, recruiting circulating progenitors, and inhibiting cells apoptosis. Altogether, through these indirect mechanisms, the therapeutic cells accelerate the formation of the vascular network and enhance healing processes [61].

Nature of EPC: the precise definition of EPCs remains uncertain and controversial. In general, they are characterized by the coexpression of markers for both hematopoietic and endothelial cell lineages (CD34, CD133, VEGF receptor-2, kinase-insert domain receptor, von Willebrand factor, and endothelial nitric oxide synthase) [62]. CD133+ cells can be considered as EPC progenitors. When CD133 expression decrease there is an increased expression of a variety of endothelial lineage markers constituting a signal for EPCs maturation toward the endothelial lineage [63]. Expression of such markers can distinguish between early EPCs (e.g., CD133+ CD34+ cells) and late-outgrowth EPCs. To note that while the subpopulation of late-outgrowth EPCs form vascular networks de novo and can incorporate into nascent blood vessels, early EPCs indirectly augment vasculogenesis via the paracrine mechanism [61,62,63,64].

Although it has been shown number of administered CD34+ cells has to influence cellular therapy’s clinical outcome [65,66]. Other studies, Ruiz-Salmeron [30] and the (PROVASA) study—Intraarterial Progenitor Cell Transplantation of Bone Marrow Mononuclear Cells for Induction of Neovascularization in Patients with Peripheral Arterial Occlusive Disease trial do not reach this conclusion [67].

Despite the controversial results reported with CD34+ cells, BM-MNCs and MSC there are enough data to support a retrospective study on the long-term effects of cell therapies in CTLI. Table 9 shows long-term effects of CD34+ and BM-MSC on leg amputations.

## 3. Discussion

### 3.1. Autologous vs. Allogenic ATMPs

ATMPs come in two main categories: allogenic and autologous. Allogenic ATMPs are obtained from one healthy donor and then expanded allowing to treat multiple patients. In contrast, in autologous therapies, the biological material originates from the patient to be treated, undergoes modification, and is then expanded for the treatment of the same patient.

HLA-matching, extremely critical in bone-marrow stem cell transplantation and other tissues and organ transplants, is not when MSC are used. MSC are considered to be immune-privileged since they express low levels of HLA Class I and no HLA class II, and therefore do not activate lymphocytes. Giving we identify the best donor are the optimal ATMP (Table 10).

In 2019 [69] we proposed that an optimal ATMP may come from a combination among the following factors:

(a) A new source of healthy allogeneic donors. In the NOMA Project we used healthy young female altruistic donors of adipose tissue [69].
(b) A cost-effective mass production under GMP conditions (to be developed).
(c) A safe, friendly and less costly procedure for administration, for example, via the intramuscular route. Described in the NOMA Project [69].
(d) A new xeno-free culture medium. In fact, a xeno-free and human-component free has been developed by B. Soria (patent pending).

Autologous stem cell transplants are custom-made exclusively for the recipient, who is also the donor. However, this personalized approach may be used in genome editing of a point gene mutation, but with may not be feasible when a patient’s own cells are affected by their own condition’s systemic inflammation [41].

In a more recent study, Carvalho and collaborators have characterized human MSC from old (60–80 years old) and young (30–45 years old) healthy donors by flow cytometry and multilineage differentiation assays and discovered that when it comes to cell proliferation, there is a notably higher rate of cell division in cells obtained from younger donors compared to those from elderly donors. It has been observed that after seven days of culture, there is a significant increase in the number of cells from young MSC in comparison to old MSC [70], suggesting that the ability of MSC to proliferate in vitro decreases as the age of the donor increases. Furthermore, MSC from elderly donors shown decreased differentiation potential as well as decreased proliferation capacity [71,72]. Overall, these characteristics potentially jeopardize the practicality of autologous MSC-based treatments in geriatric patients since aged MSC have a senescent phenotype, which has an impact on clinical results since transplanting MSC with a high proportion of senescent cells is less effective [73,74].

### 3.2. Adverse Effects of Cell Therapy with ATMP

#### 3.2.1. Bone-Marrow Mononuclear Cells (BM-MNC)

Potential risks associated with ATMP cell therapy administration may include a progression of renal failure (by increased rhabdomyolysis), diabetic retinopathy (Proliferation due to VEGF), cardiovascular risk, and possible cancer potentiation or acceleration. However, most studies coincide in no-adverse effects of BM-MNC cell therapy, either intraarterial or intramuscular [75,76]. Even more, no relationship between administration of cell therapy and an increased risk of cancer incidence has been demonstrated [77,78]. 

#### 3.2.2. Autologous MSC

Unexpectedly, in the MSC trial NCT0125776, two diabetic patients developed peripheral microthrombosis [41]. This adverse effect, which contrasts with the reported antithrombotic properties of MSCs [79], may be due to the diabetic environment that alters the fibrinolytic activity of MSCs, thereby increasing the probability of developing thrombosis. This premise was furtherly confirmed by demonstrating that diabetic MSCs cultured in the presence of blood sera expressed and released higher levels of plasminogen activator inhibitor type 1, reduced levels of tissue plasminogen activator, and lower D-dimer formation compared with nondiabetic MSCs. Thus, to establish an appropriate cell therapy for diabetic patients, we recommend including new preclinical safety tests, such as the D-dimer and/or the tissue plasminogen activator-to-plasminogen activator inhibitor type 1 ratio tests, to assess fibrinolytic activity of cells before implantation [80].

**Figure 9:**
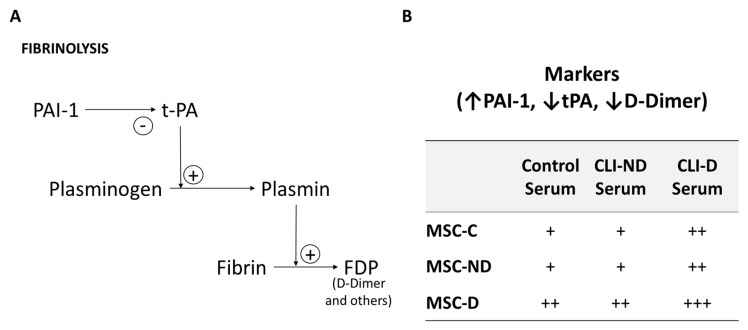
(**A**) Illustrative diagram of the involvement of some of the factors that participate in fibrinolysis and thrombosis. (**B**) The excess or deficiency of any of the factors of the fibrinolysis-antifibrinolysis system can induce hemorrhage or thrombosis. PAI−1: Plasminogen Activator Inhibitor−1; t−PA: tissue Plasminogen Activator; FDP: Fibrinogen Degradation Products. MSC−C; Control Mesenchymal Stromal Cells. MSC-ND; Non-Diabetic patients Mesenchymal Stromal Cells. MSC−D; Diabetic patients Mesenchymal Stromal Cells. CLI−ND; Non-Diabetic Critical Limb Ischemia. CLI−D; Diabetic Critical Limb Ischemia.

Figure 9 represents the involvement of some of the factors involved in fibrinolysis and thrombosis. For this, control MSCs (MSCs-C), MSCs from diabetic patients (MSCs-D) and non-diabetic (MSCs-ND) in the presence of commercial serum (control), diabetic (CLI-D) and non-diabetic (CLI-ND) human serum. Under control conditions, through qPCR analysis of the three populations cells obtained similar levels of expression of tPA, obtaining a significant decrease (*p* ≤ 0.05) in MSCs-ND with respect to MSCs-C at the secreted protein level. When stimulated with human serum (CLI-Control, CLI-D and CLI-ND), the levels of both. Both transcriptional and secreted protein of tPA were significantly lower in MSC-D (*p* ≤ 0.01) with respect to MSCs-C, suggesting that the addition of serum, regardless of its origin, inhibits the expression of tPA in these cells.

Likewise, PAI-1 levels were evaluated. It has been shown that an excess of this inhibitor of the fibrinolytic system promotes the pathological deposition of fibrin favoring thrombotic events. The transcriptional levels of PAI-1 were significantly higher in MSCs-D in all culture conditions with respect to the MSCs-ND. The MSCs-C presented an expression pattern similar to MSCs-D. However, regarding the amount of protein released, MSCs-D secreted the highest amount of PAI-1, reaching 400 ng/mL in the presence of serum from diabetic people (CLI-D), while the MSCs-C and the MSCs-ND, regardless of culture conditions, released between 150 and 200 ng/mL of protein. In the presence of the control serum MSCs-C and the MSCs-D secreted similar levels of PAI-1, while MSCs-ND did in less quantity (Figure 9B).

**Figure 10:**
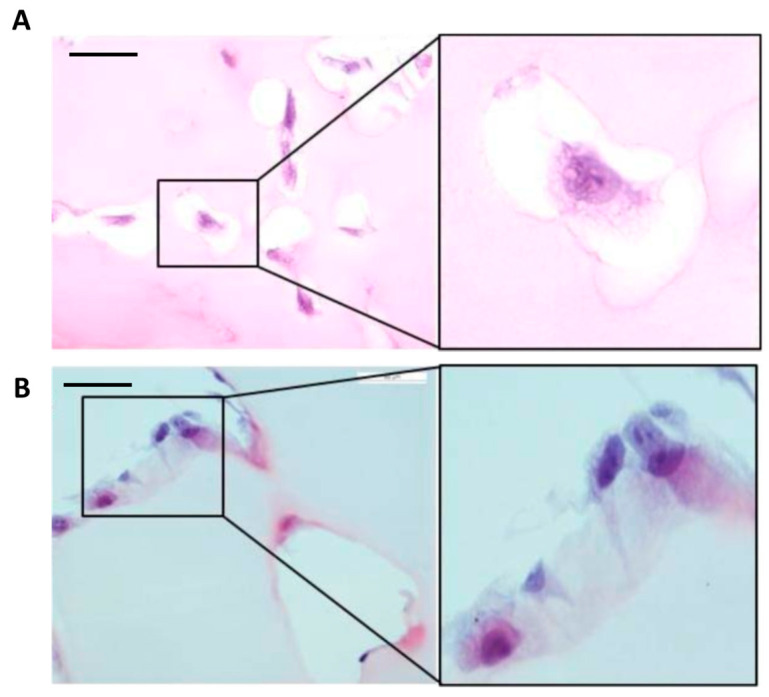
Degradation of fibrin gel. Eosin-hematoxylin staining (**A**) Gel degradation of the fibrin by control cells (MSC-C). Scale bar: 50 μm (**B**) Degradation of fibrin gel in MSC-D. Diabetic cells have attenuated their fibrinolytic capacity. MSC-C; Control Mesenchymal Stromal Cells. MSC-D; Diabetic Mesenchymal Stromal Cells. Scale bar: 50 µm.

To mimic an ex vivo fibrinolytic situation, the MSCs were cultured in fibrin gels with control serum and in the presence of different sera human blood cells (control, -D and -ND). The degradation of the fibrin gel was found to be dysregulated (decreased) in MSCs-D, regardless of the spiked human blood serum (Figure 10), as observed by lysis of the gel around the cells. This effect occurred regardless of the established growing conditions. In order to validate this observation, the production of D-dimer (DD) by the MSCs was quantified by culture in fibrin gels. DD is produced after the degrading action of plasmin on fibrin during fibrinolysis. Therefore, it is possible to find high values of DD with an increase in fibrinolytic capacity. The quantification of the DD gave as results values decreased with respect to the control, which is related to a greater prothrombotic risk.

**Figure 11:**
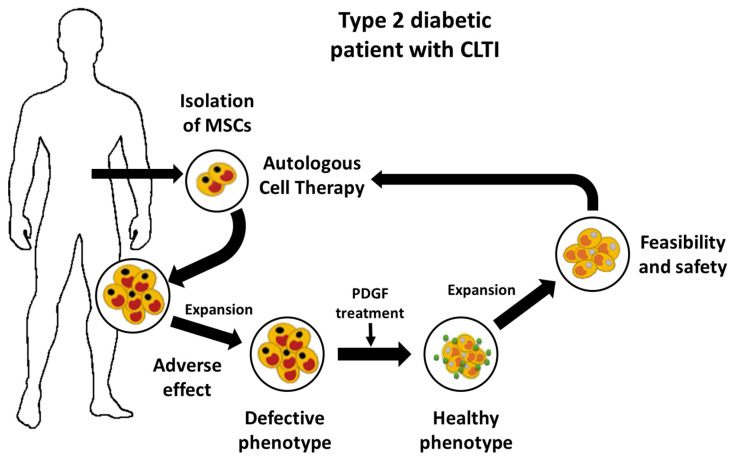
Platelet-Derived Growth Factor (PDGF) restoration of Defective Phenotype of Adipose-Derived Mesenchymal Stromal Cells from Type 2 CTLI Diabetic Patients. PDGF: Platelet-Derived Growth Factor. MSC: Mesenchymal Stromal Cells (modified from Capilla-González et al. [81]).

Here, we found that MSCs derived from diabetic patients with CLI not only exhibit a prothrombotic profile but also have altered multi-differentiation potential, reduced proliferation, and inhibited migration and homing to sites of inflammation. We further demonstrated that this aberrant cell phenotype is reversed by the platelet-derived growth factor (PDGF) BB, indicating that PDGF signaling is a key regulator of MSC functionality. These findings provide an attractive approach to improve the therapeutic efficacy of MSCs in autologous therapy for diabetic patients (Figure 11).

##### Ulcers, Amputations and QALY: Cost-Effectiveness of Treatments

Quality-adjusted life years (QALYs) have been used to evaluate new treatments for DFU. The primary outcomes are the cost (savings) per patient without a foot ulcer (i.e., cost-effectiveness) and per QALY gained (i.e., cost-utility).

The cost of treating CLI is quite high, being almost $30,300. The incremental cost-effectiveness ratio (ICER) of endovascular revascularization was €38,247.41/QALY (∼$50,869/QALY). For the United States it has been estimated that the cost of care for CLI patients was $4 billion annually in the in 2007. A comparative study for elderly patients unsuitable for surgery it was found that endovascular revascularization was cost-effective compared with conservative treatment. For example, endovascular revascularization was cost-effective compared with conservative treatment with an incremental cost-effectiveness ratio (ICER) of €29,898.36/QALY (∼$39,765/QALY) and €56,810.14/QALY (∼$75,557/QALY), respectively. Recovery following revascularization for CTLI may take weeks to months, and sometimes patients never return to their gainful employment, which has a quantifiable cost. Obviously, these figures are not so high in Europe, however the cost of treating CLI increases with disease severity, and the inpatient cost of treating intermittent claudication (IC) in 2015 was $19,300, while the cost of treating CLI was almost $30,300. In conclusion, an intramuscularly applied ATMP with a manufacture cost about 2000–3000 $/€ will be 10 times cheaper and 60% more effective than the Standard of Care (SoC).

##### Responders and Non-Responders

Given the differences observed in the therapeutic response of ATMPs for patients with no-option limb ischemia the point of which are the characteristics for responders and non-is crucial. In those patients treated with bone-marrow mononuclear cells those youngers, with low C-protein and high TcPO_2_ responded better, furthermore higher CD34+ concentration in the cellular medicament is an independent variable for better response [82], and observation that seems to be in contradiction with the effect of mesenchymal stromal cells in which as higher the inflammation as bigger the effects. Based in previous attempts for cardiovascular disease cell therapy in which a combination of factors: genetic, ethnic, age, sex, etc. may feed artificial intelligence algorithms in order to find the best candidates for cell therapy [83].

##### MSC Origin and Effect

Comparative studies between two diabetes patient groups have repeatedly demonstrated that bone marrow mesenchymal stem cells (BM-MSC) transplantation may provide better results than bone marrow-derived mononuclear stem cells (BM-MNCs) on a number of different criteria. Angiogenesis scores, Ankle-Brachial Index (ABI), transcutaneous oxygen tension (TcPO_2_), rest pain, and painless walking time all seem to improve better when BM-MSCs are used. According to these results, BM-MSC transplantation may be able to improve blood circulation in the lower extremities and reduce pain in diabetics who have critical limb ischemia (CLI) [84,85,86,87].

## 4. Material and Methods

### 4.1. Neovascularization

For each patient, a comparison was made of the images obtained by digital substraction angiography of the target lower limb taken before, 6 months and 12 months after cell infusion. Image acquisition was performed by a vascular fluoroscopic imaging system (Infinix, Toshiba, Japan) and focused on the infrapopliteal region. The analysis of neovascularization was blinded using a software validated previously [30].

i. Flow cytometry.

Aliquots of freshly isolated cells resuspended in cold PBS-2% BSA-2.5mM EDTA solution were incubated with antibodies for 30 min on ice. Stained cells were then fixed with 4% paraformaldehyde and then analysed on a FACSCalibur flow cytometer (BD Biosciences, Madrid, Spain). Data were analysed using Flowing Software version 2.5.1 (Turku Centre for Biotechnology, Univ. Turku, Finland).

### 4.2. Ulcer Healing

The ulcers were measured taking as reference the largest diameter of the wound and the smallest diameter, and applying the elliptical method described by Shaw and collaborators [88]. Healing of ulcers on the target limb. Ulcers were classified according to the University Classification System of Texas proposed by Armstrong, Lavery and Harkelss [89,90]. It was the first two-dimensional classification, where lesions are staged based on two main criteria: depth and existence of infection/ischemia. The longitudinal axis of the matrix deals with the parameter depth with four degrees (from grade 0 to grade 3) and the vertical axis is deals with the infection/ischemia parameter, with the assignment of four letters (from A to D).

### 4.3. Pain and Walking Capabilities

Pain and walking capability in patients affected by peripheral arterial ischemia are the symptoms by which most patient go to medical consultation and through which the diagnosis. Pain was measured according to the changes produced in the intensity of pain by the patient during the study using the visual analog scale of pain (VAS scale). This scale consisted of the patient marking the intensity of the pain on a scale from 0 to 10, with 0 being considered “no pain” and 10 being “unbearable pain.”

### 4.4. Transcutaneous Oxygen-TcPO_2_

TcPO_2_ measurement analyzes microcirculation and tissue perfusion after diffusion of oxygen from the vessels. Measurements were performed in the patient’s supine position using an oxygen monitor (TCM400 Radiometer, Copenhagen, Denmark). Interpretation of TcPO_2_ values: Low values < 20 mm Hg; Average values: between 20–40 mm Hg; Normal values: >40 mm Hg.

### 4.5. Ankle-Arm Index (ABI)

The changes produced during the study in ABI values were evaluated for all patients. The index, in cases where it could be quantified, was calculated using the following formula:

ABI = Systolic pressure in the pedia or the tibial/systolic pressure in the brachial artery

The ABI values were presented as follows: Low ABI: values less than 0.9 were considered normal ABI: values between 0.91 and 1.2 and abnormal ABI: values greater than 1.2.

### 4.6. Rutherford-Becker Scale

According to the degree of severity of the disease, all patients were classified according to the RB Scale (see Table 5) in grade II category 4 (R II-4, those who experience pain at rest) or in grade III-categories 5 -6 (R III-5-6, classification related to the presence of trophic lesions). This scale takes into account both hemodynamic and clinical criteria. In the treatment groups, both in experimental group 1 and in experimental group 2, there was a higher proportion of patients with tissue loss (grade III) than in the control group (40% vs. 20%).

### 4.7. Amputations

A review of the patients’ medical records was carried out to collect data on amputations prior to the study. Likewise, amputations were noted that took place during the course of the study, differentiating between majors and minors. Major amputations were considered those performed at the level of the ankle joint (Syme’s), at the middle level of the tibia (infracondylar or transtibial), at the level of the knee joint, the one made above the knee (supracondylar) or at the level of the hip. Transphalangeal, digital metatarsal and transmetatarsal amputations of the foot were considered minor amputations.

## 5. Conclusions

As in CTLI the primary objective remains to be the augmentation of angiogenesis and MSCs secrete a host of paracrine angiogenic and immunomodulatory factors [91] and considering the evolution of the process, the time-window for CTLI therapy with MSC extends from the onset of the intermittent claudication to the observation of small ulcers or non-revascularizable disease. This early administration of ATMPs (as shown in Figure 3, left of the dash line), may be the best treatment opportunity for these patients hence can significantly prevent limb amputation. Patients whose complications exceed this point may benefit from Compassionate Use, innovative treatments such as photodynamic therapies for infected ulcers or conventional surgery.

## 6. Patents

- Bernat Soria, Abdelkrim Hmadcha, Lourdes Acosta, Natalia Escacena (2012) “Method for predicting treatment response and test for safe use of mesenchymal stem cells on inflammatory diseases” PCT/EP2014/066600
- Soria Escoms, B. (2020) “Safe and Effective Pharmaceutical Products for COVID-19 and other Inflammatory, Autoimmune and Degenerative Diseases”. EP20382405 (14 May 2020).

## Figures and Tables

**Table 1:** Prevalence of Diabetes Mellitus (DM) in 2021 and projected prevalence for 2030 and 2045 [2]. In USD millions.

	2021	2030	2045	Increase (%)
Global	537	643	783	46
Europe	61	67	69	13
North-America & Caribbean	51	57	63	24
Western Pacific	206	238	260	27
South & Central America	32	40	49	50
Africa	24	33	55	134
Middle East & North Africa	73	95	136	87
South East Asia	90	113	152	68

**Table 2:** Direct Cost of Diabetes Mellitus [2].

Country	Cost (in USD Billion)
United States of America	379.5
China	165.3
Brazil	42.9
Germany	41.3
Japan	35.6
United Kingdom	23.4
France	22.7
Mexico	19.9
Spain	15.5
Italy	14.7

**Table 4:** Composition of Bone Marrow Mononuclear Cells of the cell suspension used in NCT008872326 as compared with Cord Blood and Peripheral Blood (carried out by Prof. Bernat Soria in the period 2007-2009). ND; Not Determined. HSC; Haematopoietic Stem Cells. VEGFR2; Vascular Epithelial Growth Factor Receptor 2. CXCR4; Chemokine Receptor 4.

	Bone Marrow (%)	Cord Blood (%)	Peripheral Blood (%)
**Mononuclear Cells**	24.6 to 87.4 of white blood cells	ND	ND
**Monocytes**		6.3	6.5
**Stem Cells CD34+ (Hematopoietic Stem Cells-HSC)**	1.8 to 11.5 of mononuclear cells	0.3 of mononuclear cells	
**-Early non-committed HSC (CD38−)**	8.5 to 50.4% of CD34+ cells	ND	ND
**% of Mononuclear Cells**			
**Monocytes (CD45+CD14+)**	6.3	14.1	16.4
**Lymphocytes**	23.1 to 47.5	38.3	33.2
- **B-cells (CD19+)**	15.7	11.7	8.2
- **T-cells (CD3+)**	51.6	46.8	62.8
- **Helper T cells (CD3+CD4+)**	27.4	32.2	37
- **Cytotoxic T-cells (CD3+CD8+)**	19	19.6	24.1
- **Treg Cells (CD3+CD25+)**	2.5	0.8	3.6
- **NK Cells (CD3-CD16+CD56+)**	6.3	18.2	0.86
**Dendritic cells**	1	0.88	1.4
**VEGFR2 expressing cells**	0.5 to 20.3 of White blood cells	ND	ND
**CXCR4 (Proangiogenic)**	0.4 to 9.7	ND	ND

**Table 5:** Rutherford-Becker scale patient classification [46,47].

Rutherford Classification		
Grade	Category	Symptoms
		Asymptomatic, hemodynamically unstable
**I**	1 2 3	Mild Claudication Moderate Claudication Severe Claudication
**II**	4 5	Ischemic pain at rest Ulcers, gangrene
**III**	6	Loss of tissue No function Amputation need

**Table 6:** Effects of BM-MNCs on Rutherford-Becker Categories and University of Texas Ulcer Scale time-course in CLI diabetic patients (*n* = 20). Values in bold indicate a clear improvement rate in high % of the population (taken from Ruiz-Salmeron et al., 2011, with permission [30]).

			Baseline Cases (%)	3 Months Cases (%)	12 Months Cases (%)
**Rutherford- Becker**	**Categories**	Cat 0		0	4 (25%)
		Cat 1		5 (26.4%)	9 (**56.25%**)
		Cat 2		12 (**63.1%**)	3 (18.75%)
		Cat 3			
		Cat 4	3 (15%)		
		Cat 5	11 (55%)	2 (10.5%)	
		Cat 6	6 (30%)		
**ULCERS** **University of Texas**	**Stage A**	No Ulcer	1 (5%)	-	-
		A0	3 (15%)	15 (**79%**)	14 (**87.5%**)
		A1		2 (10.5%)	1(6.25%)
		A2	1 (5%)	1 (5.3%)	
		A3			
	**Stage C**	C0	3 (15%)		
		C1	3 (15%)		
		C2	3 (15%)		
		C3	6 (30%)		

**Table 7:** Number of patient-cases (%) classified according to the severity of the disease using the Rutherford-Becker scale throughout the trial (taken from Escacena, 2016 [35]).

	RB Grade	Basal Cases (%)	1 Month Cases (%)	3 Months Cases (%)	6 Months Cases (%)	9 Months Cases (%)	12 Months Cases (%)
Control	0					1 (10%)	1 (10%)
	I			1 (10%)	2 (20%)	2 (20%)	2 (20%)
	II	8 (80%)	7 (70%)	7 (70%)	5 (50%)	2 (20%)	6 (60%)
	III	2 (20%)	3 (30%)	-	1 (10%)	5 (50%)	1 (10%)
	Total	10	10	8	8	10	10
Exp. Group 1	0				3 (30%)	3 (30%)	4 (40%)
	I		4 (40%)	4 (40%)	1 (10%)	1 (10%)	1 (10%)
	II	5 (50%)	1 (10%)	2 (20%)	2 (20%)	2 (20%)	2 (20%)
	III	5 (50%)	5 (50%)	4 (40%)	4 (40%)	4 (40%)	3 (30%)
	Total	10	10	10	10	10	10
Exp. Group 2	0		1 (10%)	5 (50%)	4 (40%)	6 (60%)	6 (60%)
	I		3 (30%)	3 (30%)	3 (30%)	2 (20%)	3 (30%)
	II	6 (60%)	4 (40%)	-	-	-	-
	III	4 (40%)	1 (10%)	1 (10%)	2 (20%)	2 (20%)	1 (10%)
	Total	10	9	9	9	10	10

**Table 8:** Amputations in CTLI patients that have received MSC. Follow-up 12 months [35].

	Control	Exp. Group 1	Exp. Group 2
6 Months	Patients (%)		
Patients with Amputation	10% (1)	20% (2)	10% (1)
Patients with major amputation	0%	0%	0%
Total Amputations	2	3	4
12 Months	Patients (%)		
Patients with Amputation	10% (1)	10% (1)	10% (1)
Patients with major amputation	0%	0%	0%
Total Amputations	2	1	1

**Table 9:** Amputations with AMTP therapy. Modified from Lara-Hernández, 2015 [68] and B. Soria (unpublished data). SoC; Standard of Care. MSC; Mesenchymal Stromal Cells. BM-MSC; Bone-Marrow Mesenchymal Stromal Cells.

	1 Year	2 Years	5 Years	10 Years	20 Years
SoC	81.6	92.1	100	100	100
SoC + CD34+	23.2	36.4	41.9	NA	NA
SoC + BM-MSC	8	10	25	26	40

**Table 10:** Autologous and allogeneic ATMPs. HLA: Human Leukocyte Antigens, GMP: Good Manufacture Practice.

Source	HLA Matching	GMP Manufacture	Off-the-Self Pharmaceutical Product
Autologous	High	Expensive	No
Allogeneic	Low	Affordable	Yes

## Data Availability

Data is contained within the article.
